# Supplementary figures and images for: Descriptive anatomy of the largest known specimen of Protoichthyosaurus prostaxalis (Reptilia: Ichthyosauria) including computed tomography and digital reconstruction of a three-dimensional skull
Source: PeerJ. 2019 Jan 8;7:e6112. doi: 10.7717/peerj.6112 (PMC6329338; doi:10.7717/peerj.6112)

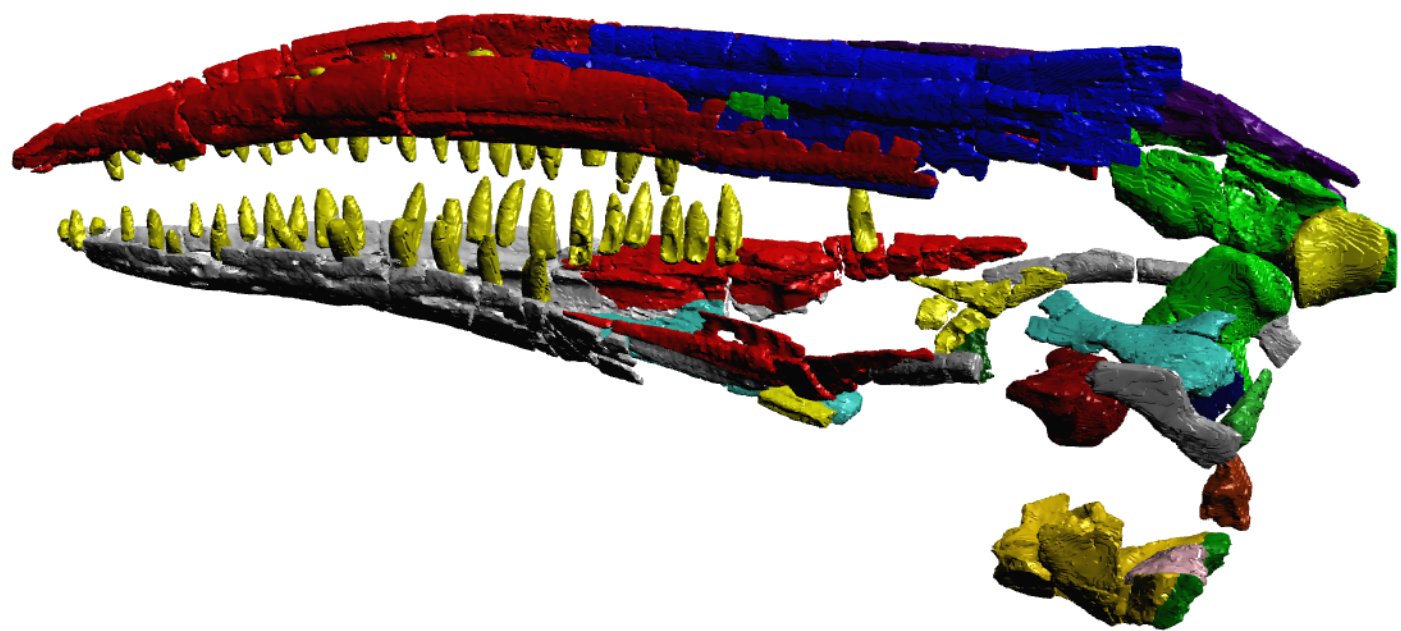

Supplement: Supplemental Information 1 — Download the PDF file and click once on the skull to activate. Left-click to rotate the model; right-click to zoom in or out; and hold both buttons to pan. Check or uncheck boxes in the model tree in the upper left corner of the viewer to display or hide individual parts. [file peerj-07-6112-s001.pdf]

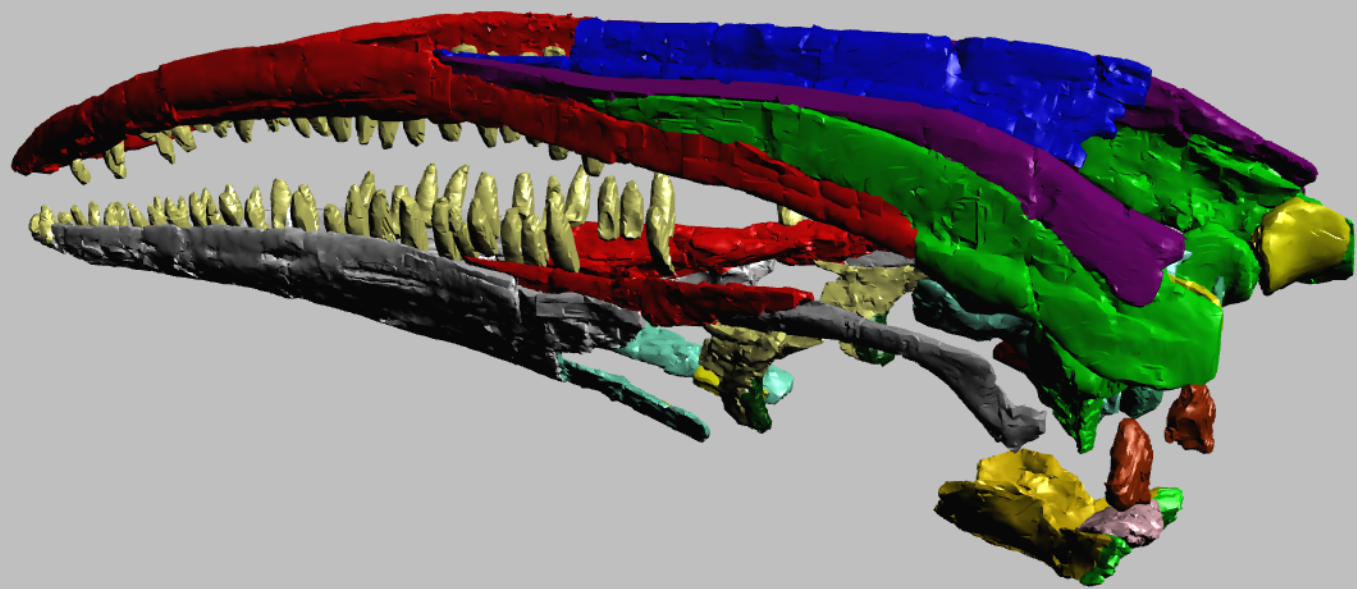

Supplement: Supplemental Information 3 [file peerj-07-6112-s003.pdf]
