# Supplementary material for: Descriptive anatomy of the largest known specimen of Protoichthyosaurus prostaxalis (Reptilia: Ichthyosauria) including computed tomography and digital reconstruction of a three-dimensional skull
Source: PeerJ. 2019 Jan 8;7:e6112. doi: 10.7717/peerj.6112 (PMC6329338; doi:10.7717/peerj.6112)
Supplement: Supplemental Information 2 [file peerj-07-6112-s002.docx]

**Appendix S2.** Matrics to transform original CT data set to reconstructed 3D skull in Avizo.

Anterior half of snout to posterior half

-0.999529 -0.0306322 0.00113535 0 -0.0306529 0.998998 -0.032456 0 -0.00014001 -0.0324756 -0.999473 0 -8.74252 -8.27617 -885.501 1

Fragment of pterygoid to pterygoid

0.98753 0 -0.157435 0 0 1 0 0 0.157435 0 0.98753 0 2.35189 -1.22478 14.7396 1

Left splenial to left dentary

0.894233 -0.446405 0.0326731 0 0.447562 0.892705 -0.0525249 0 -0.00572027 0.0615929 0.998084 0 -47.3794 71.5752 4.90189 1

Posterior left splenial to anterior left splenial and left dentary

0.877727 -0.479161 0.000343159 0 0.47105 0.862999 0.182595 0 -0.0877881 -0.160106 0.983185 0 -68.4793 19.493 -25.0443 1

Left articular to left surangular

0.965562 0.168498 -0.198239 0 -0.170395 0.985346 0.00757469 0 0.19661 0.0264651 0.980124 0 23.8269 -13.7904 17.3451 1

Whole right lower jaw – move 1

-1 0 0 0 0 1 0 0 0 0 1 0 -20.9972 0 0.489594 1

Front left upper jaw to same anterior level as lower jaw

1 0 0 0 0 1 0 0 0 0 1 0 0 -0.315235 -27.9457 1

Left lacrimal to left maxilla

0.96239 0.252805 0.0994593 0 -0.258643 0.964637 0.0507875 0 -0.0831028 -0.0746019 0.993743 0 -25.37 -40.4928 -53.046 1

Left jugal to left maxilla and lacrimal

0.996823 0 0.0796363 0 0 1 0 0 -0.0796363 0 0.996823 0 -36.7452 1.1791 -72.6955 1

Left quadrate to lower jaw joint

0.964084 1.95578e-008 -0.265593 0 0.0878615 0.943696 0.318932 0 0.250639 -0.330812 0.909804 0 23.9275 -27.4717 19.4973 1

Left pterygoid to quadrate

0.964821 0.246355 -0.0918238 0 -0.2474 0.968913 -3.72529e-009 0 0.0889693 0.0227172 0.995775 0 4.87755 19.4105 0.683945 1

Basispenoid to left pterygoid

0.987382 0 0.158359 0 0 1 0 0 -0.158359 0 0.987382 0 -60.8868 22.4298 16.4626 1

Basisphenoid – move 2

0.993654 0 0.11248 0 0 1 0 0 -0.11248 0 0.993654 0 -56.2295 22.4298 21.7633 1

Left stapes to quadrate and pterygoid

0.942366 0.303973 -0.139806 0 -0.300681 0.95268 0.044608 0 0.14675 3.35276e-008 0.989172 0 -16.3699 26.7368 8.56757 1

Left opisthotic to stapes

1 0 0 0 0 1 0 0 0 0 1 0 -16.8867 39.7966 2.83831 1

Left parietal/supratemporal/supraoccipital to *approximate* vertical position

1 0 0 0 0 1 0 0 0 0 1 0 -33.4553 24.1336 0 1

Extracted right side of skull

0.994062 0 0.108817 0 0 1 0 0 -0.108817 0 0.994062 0 -151.868 0 -13.7749 1

Slight adjustment for whole left lower jaw

0.999686 0 0.0250459 0 0 1 0 0 -0.0250459 0 0.999686 0 -10.4185 0 -6.26843 1

Whole right lower jaw – move 2

0.996397 0 0.0848097 0 0 0.999999 0 0 -0.0848097 0 0.996397 0 -63.1146 -1.52588e-005 -12.6747 1
